# Appropriate feeding practices and associated factors during diarrheal disease among children aged 6 to 23 months in Sub-Saharan Africa: a multilevel analysis of the recent demographic and health survey

**DOI:** 10.1186/s12887-023-04480-6

**Published:** 2024-01-06

**Authors:** Belayneh Shetie Workneh, Enyew Getaneh Mekonen, Alebachew Ferede Zegeye

**Affiliations:** 1 Department of Emergency and Critical Care Nursing, School of Nursing, College of Medicine and Health Sciences, University of Gondar, Gondar, Ethiopia; 2 Department of Surgical Nursing, School of Nursing, College of Medicine and Health Sciences, University of Gondar, Gondar, Ethiopia; 3 Department of Medical Nursing, School of Nursing, College of Medicine and Health Sciences, University of Gondar, Gondar, Ethiopia

**Keywords:** Africa, Children, Diarrheal Disease, Sub-saharan, Appropriate feeding

## Abstract

**Background:**

Diarrhea is the second leading cause of morbidity and mortality for under-five children which cause about 525,000 deaths annually. Even though diarrheal diseases have decreased substantially at the global level, low-income countries are still faced with a huge number of diarrheal diseases. Thus, our aim was to assess the child feeding practices during diarrheal diseases and associated factors among children aged 6 to 23 months in Sub-Saharan African countries using the recent demographic and health survey.

**Methods:**

The appended and most recent demographic and health survey (DHS) dataset of 19 Sub-Saharan African countries from 2015 to 2020 was used for data analysis. A total of 64,628 living children aged 6–23 months with diarrhea were used as a weighted sample. The determinants of appropriate feeding practice were determined using a multilevel mixed-effects logistic regression model. Significant factors associated with appropriate feeding practice in the multilevel mixed-effect logistic regression model were declared significant at *p*-values < 0.05. The adjusted odds ratio (AOR) and confidence interval (CI) were used to interpret the results.

**Result:**

The overall prevalence of appropriate child feeding practice during diarrhea in this study was 6.24% (95% CI: 6.06, 6.43). Maternal age (15 to 19 years and 20 to 35 years) (AOR = 1.32, 95%CI: 1.12, 1.55 and AOR = 1.14, 95%CI: 1.03, 1.27), mothers education (primary and secondary level) (AOR = 1.23, 95%CI: 1.12, 1.35 and AOR = 1.28, 95%CI: 1.15, 1.43), having media exposure(AOR = 1.36, 95%CI: 1.26, 1.46), being married (AOR = 1.18, 95%CI: 1.01, 1.38), currently working (AOR = 1.08, 95%CI:1.00, 1.15), vaccinated for Rotavirus (AOR = 1.30, 95%CI:1.19, 1.43) and living in Central and eastern African countries (AOR = 1.82, 95%CI: 1.12, 2.97) and (AOR = 2.23, 95%CI: 1.37, 3.61) respectively were significantly associated with appropriate feeding practice.

**Conclusion:**

The prevalence of appropriate feeding practice during child diarrheal disease aged 6–23 months of age was strictly low which implies that child diarrhea and appropriate feeding practice is still a great issue in in Sub-Saharan African countries. Enhancing maternal education, strengthening media exposure and vaccination for rotavirus, and designing interventions that address the mother’s marital status, mother’s work status, and country category are recommended to enhance appropriate feeding practices. Furthermore, special consideration should be given to older mothers to increase appropriate feeding practices during diarrheal disease.

## Introduction

Diarrhea is the second leading cause of morbidity and mortality for under-five children which causes about 525,000 deaths and nearly 1.7 million cases reported annually [1]. The report of UNICEF reveals diarrhea is a leading cause of death for under-five children which accounts for approximately 9% of all the deaths among children under-five worldwide. Globally 1,300 young children die every day despite the availability of a simple treatment [2].

Even though diarrheal diseases have decreased substantially at the global level, low-income countries still face a huge number of diarrheal diseases, due to low socioeconomic status, and inadequate access to clean water, sanitation, and hygiene [3]. Children in sub-Saharan Africa are more than 15 times more likely to die before the age of five than children in developed countries [4].

If childhood diarrhea is not managed appropriately, it causes complications like infectious diseases, malnutrition, growth, and mental retardation [5]. World Health Organization (WHO) and the United Nations for International Children Emergency Fund recommend continued feeding and fluid replacement as an intervention for diarrhea at the home level by the caregivers to reduce the complications [6]. Despite the recommendation of the WHO and UNICEF, the overall appropriate feeding practice is low in Sub-Saharan African countries [7]. A study done in Ethiopia based on the Ethiopian Demographic and Health Survey 2016 showed that the overall good feeding practice is 15.4% [8].

The findings of previous studies revealed that having media exposure and higher household wealth index [7], higher mothers’ educational level [7, 9], mothers age (25–34 years) and agricultural occupation of the mother [8], current breastfeed [9, 10], vaccinated for rotavirus [9], and small family size [10] are associated with the appropriate child feeding practice during diarrheal disease.

Although the provision of frequent fluid and solid foods during diarrheal disease is important to manage the diseases [1], in Sub-Saharan African countries, fluid and food curtailment among children during diarrhea is strictly high [11] and only 35% of under-five children with diarrhea get appropriate fluid replacement during diarrheal episodes in the region. Thus, this study aimed to assess the child feeding practices during diarrheal diseases and associated factors among children aged 6 to 23 months in sub-Saharan African countries using the recent DHS.

## Methods

### Study setting, period, design, and source

We have used the appended and the recent demographic and health survey (DHS) dataset of 19 sub-Sahara African countries, conducted from 2015 to 2020 to assess the prevalence and factors associated with appropriate child feeding practice during diarrheal disease in the region with multilevel analysis. DHS is a community-based crossectional study conducted every five years, to examine health and health-related indicators.

### Study population and sampling technique

The appended data from the most recent DHS surveys of 19 sub-Sahara African countries (Angola, Benin, Burundi, Cameron, Ethiopia, Gambia, Guinea, Liberia, Mali, Malawi, Nigeria, Rwanda, Serra Leone, Senegal, Tanzania, Uganda, South Africa, Zambia, Zimbabwe) were used for data analysis to determine the prevalence of appropriate feeding practice and associated factors during diarrheal disease among children aged 6 to 23 months in the Sub-Saharan African countries. The survey for every country contains different datasets, including those for males, females, children, births, and households. DHS deploys a stratified two-stage cluster design that includes enumeration areas as the first stage and generates a sample of households from each enumeration area as the second stage. Liquids given (more than usual, same as usual, somewhat less, much less, none, or don’t know) (h38) and foods offered (more than usual, same as usual, somewhat less, much less, none, never gave food, or don’t know) (h39) from child record (KR) data set was recorded to determine the outcome variable (appropriate feeding practice). A binary logistic regression model was applied to determine the factors associated with appropriate feeding practice and reported in terms of an adjusted odds ratio (AOR) with a significance level of (95%). In the univariate analysis, at 95% confidence intervals with a *p*-value of < 0.25 was considered a candidate for the multivariable analysis of data. All variables with *p*-values < 0.05 were considered statistically significant. A total weighted sample of 64,628 participants was included in the study (Table 1).

**Table 1:** Sample size for prevalence of appropriate feeding practice during diarrheal disease among children aged 6 to 23 months in the Sub-Saharan African countries

Country	Year of survey	Weighted sample(n)	Weighted sample (%)
Angola	2015	4,446	6.88
Benin	2017/18	4,260	6.59
Burundi	2016/17	4,180	6.47
Cameron	2018	2,925	4.53
Ethiopia	2016	3,105	4.8
Gambia	2019	2,509	3.88
Guinea	2018	2,157	3.34
Liberia	2019/20	1,754	2.71
Mali	2018	3,010	4.66
Malawi	2015	5,131	7.94
Nigeria	2018	9,893	15.31
Rwanda	2019/20	2,473	3.83
Serra Leone	2019	3,021	4.67
Senegal	2019	1,902	2.94
Tanzania	2015	3,320	5.14
Uganda	2016	4,707	7.28
South Africa	2016	1,032	1.6
Zambia	2018	3,008	4.65
Zimbabwe	2015	1,795	2.78
Total sample size		64,628	100

### Study variables

#### Dependent variable

The dependent variable of this study was appropriate feeding practice during diarrheal disease. To determine the outcome of the study, liquids given (more than usual, same as usual, somewhat less, much less, none, or don’t know) (h38) and foods offered (more than usual, same as usual, somewhat less, much less, none, never gave food, or don’t know) (h39) were used. The respondents of the study were asked how much fluid was given for their child to drink during the diarrhea and the respondents responded that nothing to drink, much less, somewhat less, about the same, more, and don’t know. In addition to the amount of fluid intake the study participants were asked how much food was given to eat for their child during the episode of the diarrhea and they responded that stopped food, never gave food, much less, somewhat less, about the same, more and don’t know. In this study, those children who were given more than usual fluid and food during the diarrheal episode were considered to have appropriate feeding practices.

#### Independent variables

The independent variables of this study were individual level (age of mother (15–19 years, 20–35 years, and 36–49 years), educational level of the mother (no education, primary, secondary, and higher level), total number of children (1, 2 to 5, 6 and more), having media exposure (yes or no), household wealth index (poor, middle and rich), marital status (never married, married and ever married), currently working (yes or no, distance to health facility (big problem or not big problem), vaccination for rotavirus (yes or no), a husband education level (no education, primary, secondary and higher level), sex of household head (male or female), current breast feed (yes or no), sex of child (male or female)) variables and community level (residence (urban or rural), community media exposure (low or high), community illiteracy (low or high), community poverty (low or high), country category (central, west, east and south)) variables.

### Data processing and statistical analysis

The data that were obtained from the most recent DHS data sets were cleaned, recorded, and analyzed using STATA version 14 statistical software. The DHS data’s variables are organized in clusters, and those in a cluster are more similar to one another than those of other clusters. To employ a standard logistic regression model, the assumptions of independent observations and equal variance across clusters were broken. This suggests that using a sophisticated model to take into account between-cluster factors is necessary. Given this, multilevel mixed-effects logistic regression was used to determine the factors associated with appropriate feeding practice. Multilevel mixed effect logistic regression follows four models: the null model (outcome variable only), model I (only individual-level variables), model II (only community-level variables), and model III (both individual and community-level variables). The model without independent variables (null model) was used to check the variability of appropriate feeding practices across the cluster. The association of individual-level variables with the outcome variable (Model I) and the association of community-level variables with the outcome variable (Model II) were assessed. In the final model (Model III), the association of both individual and community-level variables was fitted simultaneously with the outcome variable.

### Model building

Model building for multi-level analysis: Model comparisons were done using the deviance test and log-likelihood test and the model with the highest log-likelihood ratio and the lowest deviance was selected as the best-fitted model. Moreover, multicollinearity was tested using the variance inflation factor (VIF) and we got a VIF of less than ten for each independent variable with a mean VIF of 4.61, indicating there was no significant multicollinearity between independent variables. First bi-variable multilevel logistic regression analysis was performed, and those variables with a *p*-value of < 0.05 in the multilevel mixed-effect logistic regression model were declared as significant factors associated with appropriate feeding practice.

### Random effects

Random effects or measures of variation of the outcome variables were estimated by the median odds ratio (MOR), intra-class correlation coefficient (ICC), and proportional change in variance (PCV). The intra-class correlation coefficient (ICC) and proportional change in variance (PCV) were computed to measure the variation between clusters. Taking clusters as a random variable, the ICC reveals the variation of appropriate feeding practice between clusters is computed as; ICC = VC/(VC + 3.29)×100% where VC = cluster level variance. The MOR is the median value of the odds ratio between the area of the highest risk and the area of the lowest risk for appropriate feeding practice when two clusters are randomly selected, using clusters as a random variable; MOR= 𝑒 0.95√VC.

Moreover, the PCV demonstrates the variation in the prevalence of appropriate feeding practice explained by factors and computed as; $PCV=\frac{Vnull-VC}{Vnull}\times 100\%$; where Vnull = variance of the null model. The fixed effects were used to estimate the association between the likelihood of appropriate feeding practice and individual and community-level independent variables. It was assessed and the strength was presented using an adjusted odds ratio (AOR) and 95% confidence intervals with a *p*-value of < 0.05

## Result

### Sociodemographic characteristics of the participants

A total of weighted samples of 64,628 children with diarrhea were considered in this study. More than two-thirds, 44,853 (69.40%) of the participants were rural dwellers. Nearly half 30,899 (47.81%) of the study participants had media exposure. Nearly half 30,396 (47.03%) of the respondents were poor. The majority 53,607 (82.95%) of the study participants received the Rotavirus vaccine (Table 2).

**Table 2:** Sociodemographic characteristics of the participants

Individual level variables		Weighted frequency (n)	Percentage (%)
Age of Mother	15–19 years	6,093	9.43
	20–35 years	48,533	75.10
	36–49 years	10,002	15.48
The educational level of Mother	No education	23,610	36.53
	Primary	22,167	34.30
	Secondary	16,431	25.42
	Higher	2,420	3.74
Total children ever born	1	13,914	21.53
	2–5	37,677	58.30
	6 and more	13,037	20.17
Media exposure	Yes	40,595	62.81
	No	24,033	37.19
Household wealth index	Poor	30,396	47.03
	Middle	13,054	20.20
	Rich	21,178	32.77
Marital status	Never married	5,116	7.92
	married	45,452	70.33
	Ever married	14,060	21.76
Currently working	No	22,974	35.55
	Yes	41,654	64.45
Distance to the health facility	Big problem	25,380	39.59
	Not big problem	38,730	60.41
Vaccinated for rotavirus	Yes	53,607	82.95
	No	11,021	17.05
Husband’s education level	No education	28,901	44.72
	Primary	16,538	25.59
	Secondary	14,943	23.12
	Higher	4,246	6.57
Gender of household head	Male	51,353	79.46
	Female	13,275	20.54
Currently breastfeed	Yes	48,449	74.97
	No	16,179	25.03
Gender of the child	Male	32,909	50.92
	Female	31,719	49.08
**Community level variables**			
Residence	Urban	19,775	30.60
	Rural	44,853	69.40
Community media exposure	Low	33,729	52.19
	High	30,899	47.81
Community illiteracy	Low	34,272	53.03
	High	30,356	46.97
Community poverty	Low	33,828	52.34
	High	30,800	47.66
Country category	Central	7,371	11.41
	West	28,506	44.11
	East	27,719	42.89
	South	1,032	1.60

### Model fitness and random effect analysis

A null model was used to determine whether the data supported the decision to assess randomness at the community level. Findings from the null model showed that there was a significant variation in appropriate feeding practice during diarrheal disease between communities, with a variance of 0.070472 and a *P* value of 0.000. The variance within clusters contributed 97.9% of the variation in appropriate feeding practice, while the variance across clusters was responsible for 2.10% of the variation. In the null model, there is a variation in odds of appropriate feeding practice between higher and lower risk clusters by a factor of 1.29 times. The intra-class correlation value for Model I indicated that 2.06% of the variation in appropriate feeding practice accounts for the differences between communities. Thus, with the null model, to generate Model II we have used community-level variables like community media exposure, community illiteracy, community poverty, country category, and residence. Cluster variations were the basis for 2.01% of the differences in feeding practice, according to the ICC value from Model II. In the final model (model III), which attributed approximately 4.62% of the variation in the likelihood of appropriate feeding practice during diarrhea to both individual and community-level variables (Table 3), the likelihood of appropriate feeding practice varied by 1.28 times across low and high appropriate feeding practices clusters.

**Table 3:** Model comparison and random effect analysis for appropriate feeding practices during diarrheal disease among children aged 6 to 23 months in Sub-Saharan African countries

Parameter	Null model	Model I	Model II	Model III
Variance	0.070472	0.0693216	0.0674671	0.0672162
ICC	2.10%	2.06%	2.01%	2.00%
MOR	1.29	1.28	1.28	1.28
PCV	Reference	1.63%	4.26%	4.62%
LLR	-15069.563	-14911.271	-14987.511	-14858.704
Deviance	30139.126	29822.542	29975.022	29717.408

### Prevalence of appropriate child feeding practice during diarrheal disease

**Fig. 1:**
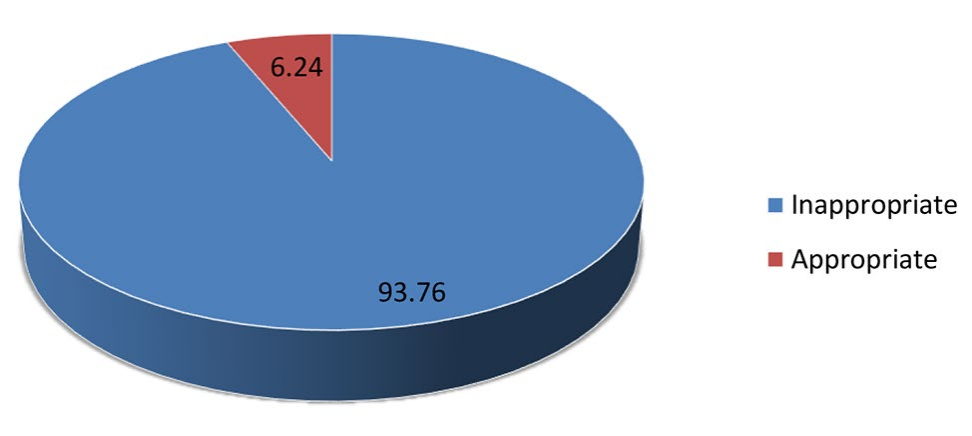
The overall prevalence of appropriate feeding practice during diarrheal disease among children aged 6 to 23 months in the Sub-Saharan African countries

The overall prevalence of appropriate child feeding practice during diarrhea in this study was 6.24% (95% CI: 6.06, 6.43) (Fig. 1). According to the findings of this study, only 3,722 children (5.76%) were given more liquid than usual, and 1,102 (1.71%) were given food more than usual. Only seven hundred ninety-two children (1.23%) were given both more fluid and food.

### Factors associated with appropriate feeding practice during diarrheal disease among children aged 6 to 23 months in Sub-Saharan African countries

From the overall variables, in the final fitted model of multivariable logistic regression, maternal age, mother level of education (primary and secondary), media exposure, marital status (married), work status, Rotavirus vaccination status, country category (central and eastern Africa) were significantly associated with appropriate feeding practice during diarrheal disease (Table 4).

The odds of appropriate feeding practice were 1.32 and 1.14 times higher among mothers aged 15 to 19 years and 20 to 35 years respectively compared with mothers aged 36 to 49 years (AOR = 1.32, 95%CI: 1.12, 1.55 and AOR = 1.14, 95%CI: 1.03, 1.27). Appropriate feeding practice was 1.23 and 1.28 more likely to be applied with mothers who have primary and secondary educational levels compared with mothers who did not have education respectively (AOR = 1.23, 95%CI: 1.12, 1.35 and AOR = 1.28, 95%CI: 1.15, 1.43). A mother who had media exposure was 1.36 times more likely to practice feeding appropriately compared to those mothers who did not have media exposure(AOR = 1.36, 95%CI: 1.26, 1.46). Those mothers who were married were 1.18 more likely to feed appropriately their child during diarrheal episodes compared with mothers who never married (AOR = 1.18, 95%CI: 1.01, 1.38). The odds of appropriate feeding practice were 1.08 times higher among mothers who currently working compared to not working (AOR = 1.08, 95%CI:1.00, 1.15). Appropriate child feeding practice was 1.30 times more likely to occur among children who received the Rotavirus vaccine compared with those who did not receive it (AOR = 1.30, 95%CI:1.19, 1.43). Central and eastern African countries were times more likely to practice feeding appropriately during diarrheal disease to less than two years of age child compared with southern African countries (AOR = 1.82, 95%CI: 1.12, 2.97) and (AOR = 2.23, 95%CI: 1.37, 3.61) respectively.

**Table 4:** Multivariable multilevel logistic regression analysis of individual-level and community-level factors associated with appropriate feeding practice during diarrheal disease among children aged 6 to 23 months in the Sub-Saharan African countries, DHS 2015–2020

Individual and community level variable	Model I AOR (95% CI)	Model II AOR (95% CI)	Model III AOR (95% CI)
Age of Mother			
15–19	1.27 (1.08, 1.49)		**1.32 (1.12, 1.55)**
20–35	1.13 (1.01, 1.26)		**1.14 (1.03, 1.27)**
36–49	1		1
The educational level of Mother			
No education	1		1
Primary	1.38 (1.27, 1.51)		**1.23 (1.12, 1.35)**
Secondary	1.33 (1.20, 1.48)		**1.28 (1.15, 1.43)**
Higher	1.01 (0.80, 1.26)		0.96 (0.76, 1.20)
Number of children			
1	0.99 (0.87, 1.12)		0.96 (0.84, 1.09)
2–5	1.01 (0.91, 1.11)		1.00 (0.91, 1.11)
6 and more	1		1
Media exposure			
Yes	1.32 (1.23, 1.42)		**1.36 (1.26, 1.46)**
No	1		1
Wealth index			
Poor	1		1
Middle	0.96 (0.88, 1.05)		0.98 (0.89, 1.07)
Rich	0.94 (0.87, 1.03)		0.95 (0.86, 1.04)
Marital status			
Never married	1		1
Married	1.18(1.01, 1.37)		**1.18 (1.01, 1.38)**
Ever married	1.15 (0.99, 1.35)		1.12 (0.96, 1.31)
Currently working			
Yes	1.07 (1.00, 1.15)		**1.08 (1.00, 1.15)**
No	1		1
Distance from health center			
Big problem	1		1
Not a big problem	0.92 (0.86, 0.99)		0.95 (0.88, 1.02)
Husband educational level			
No education			1
Primary	1.05 (0.96, 1.15)		0.94 (0.85, 1.03)
Secondary	1.00 (0.90, 1.10)		0.97 (0.88, 1.08)
Higher	0.97 (0.82, 1.14)		0.97 (0.82, 1.14)
Gender of household head			
Male	1		1
Female	1.04 (0.96, 1.14)		1.03 (0.94, 1.12)
Currently breastfeed			
Yes	1.17 (0.09, 1.27)		1.13 (0.04, 1.22)
No	1		1
Gender of the child			
Male	1.21 (0.03, 1.17)		1.10 (0.03, 1.17)
Female	1		1
Vaccinated for rotavirus			
Yes	1.20 (1.10, 1.32)		**1.30 (1.19, 1.43)**
No	1		1
**Community level Variables**			
Community media exposure			
Low		1	1
High		1.17 (1.08, 1.28)	**1.12 (1.03, 1.22)**
Community illiteracy			
Low		1	1
High		0.98 (0.91, 1.06)	0.95 (0.88, 1.03)
Community poverty			
Low		1	1
High		0.93 (0.85, 1.01)	0.94 (0.86, 1.02)
Country category			
Central		2.67 (1.75, 4.09)	**1.82 (1.12, 2.97)**
West		2.45 (1.62, 3.72)	1.53 (0.95, 2.49)
East		3.54 (2.33, 5.38)	**2.23 (1.37, 3.61)**
South		1	1
Residence			
Urban		0.98 (0.91, 1.06)	0.93 (0.85, 1.01)
Rural		1	

## Discussion

Appropriate management of diarrhea can result in minimization of mortality due to severe dehydration and other complications. Thus, investigating the prevalence and associated factors of appropriate feeding practices during diarrheal disease among children aged 6–23 months in sub-Saharan African countries is important to adapt different interventional strategies to minimize diarrhea and dehydration-related child mortality and consequences.

The finding of this study reveals that the overall prevalence of appropriate feeding during diarrheal episodes in children aged 6–23 months is 6.24% only. The result of this study showed that the majority of children aged 6–23 months do not get more than usual fluid and food during diarrheal diseases which implies that the issue of appropriate feeding practice is still a top urgent concern in sub-Saharan countries. The finding of this study is lower than the previous studies [7, 8, 12–15]. The possible reason for this variation might be due to differences in sample size, age category of the study participants, and study settings.

Coming to the associated factors, having media exposure, currently working, maternal age (15 to 19 years and 20 to 35 years), mother education (primary and secondary level), being married, being vaccinated for Rotavirus, living in Central and eastern African countries were significantly associated with appropriate feeding practice. A mother who had media exposure was 1.36 times more likely to practice feeding appropriately compared to those mothers who did not have media exposure. This finding is consistent with the previous studies [7, 16–18]. The possible explanation for this could be that women’s attitudes and knowledge can be raised by mass media through the dissemination of health-related messages. Therefore, mothers who have been exposed to the media are more likely to be knowledgeable about and to use food and fluids to manage diarrhea at the home level.

Another factor which significantly associated with appropriate feeding practice was the working status of the mother. Those mothers who are working currently had higher odds of having appropriate feeding practice compared with their counterparts. The result of this study is supported by previous studies [19, 20]. It might be due to the fact being employed reduces financial dependency. This means that mothers who are employed may not face financial constraints or difficulties in fulfilling adequate food and fluid to feed their children during diarrheal episodes compared with those mothers who are not employed.

The odds of appropriate feeding practice were 1.32 and 1.14 times higher among mothers aged 15 to 19 years and 20 to 35 years compared with mothers aged 36 to 49 years respectively which is contrary to the finding of the study [8]. It might be due to family size and educational level variation. Most of the time later motherhood is related to large family size and low level of education.

The finding of this study reveals that the odds of appropriate child-feeding practice were higher among educated mothers compared with non-educated mothers. The finding of this study is supported by the previous studies [7, 20–22]. The possible justification for this finding is that education has the potential to change the level of mothers’ knowledge. Thus education enables the mother to feed their child appropriately during the diarrheal episode.

Appropriate child feeding practice was 1.30 times more likely to be performed among caregivers whose children had received the Rotavirus vaccine compared with their counterparts. Rotavirus is a major cause of watery diarrhea in children, especially those children aged less than two years. Rotavirus vaccine is given for the prevention of diarrhea. Parents who are exposed to or have information regarding the Rotavirus vaccine have better knowledge of diarrheal disease [23]. Therefore, better knowledge of diarrheal diseases enables caregivers to manage or feed their children appropriately.

Another variable which significantly associated with the outcome variable was maternal marital status. Those mothers who were married were 1.18 more likely to feed appropriately their child during diarrheal episodes compared with mothers who never married. Married women with children do more housework compared with moms who never married [24]. Therefore, those mothers who were married spend more time at home with their children that enable them to visit, monitor, and feed their child frequently.

The geographical region was significantly associated with appropriate feeding practices. The odds of appropriate feeding practice were 1.82 and 2.23 times more likely to be practiced among mothers living in central and eastern sub-Saharan African countries compared to women living in southern sub-Saharan Africa respectively. This might be related to the difference in sociodemographic variation of the respondents.

## Conclusion

The prevalence of appropriate feeding practice during child diarrheal disease aged 6–23 months of age was strictly low which implies that child diarrhea and appropriate feeding practice is still a great issue in in sub-Saharan African countries. Enhancing maternal education, strengthening media exposure and vaccination for rotavirus, and designing interventions that address the mother’s marital status, mother’s work status, and country category are recommended to enhance appropriate feeding practices. Furthermore, special consideration should be given to older mothers to increase appropriate feeding practices during diarrheal disease.

### Limitations of the study

Important factors that could have a big impact on appropriate feeding practices, like behavior, beliefs, and social norms, are not included in the dataset. Additionally, to measure the feeding practice a social desirability bias may have been present in the mother’s verbal responses. These will hinder our findings from having the intended impact, so further studies should be carried out to explore mothers’/caregivers’ appropriate feeding practices during diarrheal disease by observing the amount and frequency of fluid and food offered to their children.

## Data Availability

The datasets generated and/or analyzed during the current study are available in the most recent data of the Demographic and Health Survey and it is publicly available online at (https://www.dhsprogram.com).
